# A Review of the In Vitro Inhibition of α-Amylase and α-Glucosidase by Chalcone Derivatives

**DOI:** 10.7759/cureus.37267

**Published:** 2023-04-07

**Authors:** Thanh-Dao Tran, Vo Linh Tu, Thai Minh Hoang, Truong Van Dat, Dao Ngoc Hien Tam, Nguyen Tuan Phat, Dang The Hung, Hong-Han Huynh, Thanh C Do, Huu-Hoai Le, Le Huu Nhat Minh

**Affiliations:** 1 Faculty of Pharmacy, University of Medicine and Pharmacy at Ho Chi Minh City, Ho Chi Minh City, VNM; 2 Regulatory Affairs Department, Asia Shine Trading & Service Company Limited, Ho Chi Minh City, VNM; 3 Faculty of Medicine, Hue University of Medicine and Pharmacy, Hue, VNM; 4 Faculty of Medicine, University of Medicine and Pharmacy at Ho Chi Minh City, Ho Chi Minh City, VNM; 5 School of Biotechnology, Tan Tao University, Long An, VNM; 6 Faculty of Medicine, Pham Ngoc Thach University of Medicine, Ho Chi Minh City, VNM; 7 International Ph.D. Program in Medicine, College of Medicine, Taipei Medical University, Taipei, TWN; 8 Research Center for Artificial Intelligence in Medicine, Taipei Medical University, Taipei, TWN

**Keywords:** endocrine, review, narrative, diabetes, chalcones, α-glucosidase

## Abstract

Diabetes mellitus is a chronic metabolic disease relating to steady hyperglycemia resulting from the impairment of the endocrine and non-endocrine systems. Many new drugs having varied targets were discovered to treat this disease, especially type 2 diabetes. Among those, α-glucosidase inhibitors showed their effects by preventing the digestion of carbohydrates through their inhibition against α-amylase and α-glucosidase. Recently, chalcones have attracted considerable attention as they have a simple structure, are easily synthesized as well as have a variety of derivatives. Some reports suggested that chalcone and its derivates could inhibit α-amylase and α-glucosidase. This narrative review provides a comprehensive evaluation of the inhibition of chalcone and its derivatives against α-amylase and α-glucosidase that were reviewed and reported in published scientific articles. Twenty-eight articles were reviewed after screening 207 articles found in four databases, including PubMed, Google Scholar, VHL (Virtual Health Library), and GHL (Global Health Library). This review presented the inhibitory effects of varied chalcones, including chalcones with a basic structural framework, azachalcones, bis-chalcones, chalcone oximes, coumarin-chalcones, cyclohexane chalcones, dihydrochalcones, and flavanone-coupled chalcones. Many of these chalcones had significant inhibition against α-amylase as well as α-glucosidase that were comparable to or even stronger than standard inhibitors. This suggested that such compounds could be potential candidates for the discovery of new anti-diabetic remedies in the years to come.

## Introduction and background

Diabetes mellitus is a chronic metabolic disorder characterized by persistent hyperglycemia resulting from endocrine and non-endocrine system impairment. Type 2 diabetes (T2D) is widely known for its serious complications on major and minor arteries, which can lead to retinal diseases, nephropathy, neuropathy, cardiovascular diseases, cerebrovascular disease, and peripheral vascular disease. According to the International Diabetes Federation's 2021 report, there were up to 6.7 million deaths due to diabetes worldwide, placing a significant burden on society. Managing diabetes and preventing its complications have become urgent concerns for health professionals [1]. T2D is a complicated disorder that can be managed with various drugs targeting specific mechanisms, including sulfonylurea, meglitinide, thiazolidinedione, and metformin. Additionally, a healthy lifestyle is also essential. α-Glucosidase inhibitors, such as acarbose, miglitol, and voglibose, are the main agents used to prevent the digestion of carbohydrates via α-amylase and α-glucosidase. These agents dramatically inhibit α-glucosidase in the intestine and moderately inhibit α-amylase in the pancreatic gland. Undigested carbohydrates then enter the colon and are broken down by bacterial enzymes, resulting in side effects such as gas, bloating, and diarrhea. The inhibitory activity against α-amylase could improve clinical outcomes by reducing adverse events of carbohydrate metabolism in the colon [2] . Chalcone is a term used to refer to a group of compounds with a 1,3-diphenyl prop-2-en-1-one scaffold. These compounds are metabolic products of land plants and are called benzalacetophenone or benzylidene acetophenone. They are initial sources for biosynthesizing flavonoids. Over the years, chalcones have attracted considerable attention due to their simple structure, ease of synthesis, and a wide variety of derivatives. These compounds are safe with numerous appreciated bioactivities, including potent anti-obesity and anti-diabetes effects [3]. The World Health Organization (WHO) estimates that by 2030, there will be 439 million people with diabetes worldwide, mainly in developing countries. Unhealthy lifestyle, sedentary habits, and a meal plan rich in refined carbohydrates and saturated fats, as well as low intake of fruits and vegetables, are contributing factors to the increased risk of developing diabetes and related health conditions such as obesity [4]. Some reports suggest that chalcone and its derivatives can inhibit α-amylase and α-glucosidase [4]. However, investigations demonstrating the inhibition of chalcones against these enzymes are limited. Therefore, this narrative review aims to provide a comprehensive evaluation of the inhibition of chalcone and its derivatives against α-amylase and α-glucosidase as reported in published scientific articles.

## Review

Search results of articles reporting inihibitive activity of chalcone against α-amylase and α-glucosidase

We initially selected *in vitro* studies published in English up to September 18th 2022, that provided information on the inhibition of chalcone and its derivatives against α-amylase and/or α-glucosidase. The search was conducted on the following four databases: PubMed, Virtual Health Library (VHL), Global Health Library (GHL), and Google Scholar, using the search terms given in Table 1.

**Table 1:** Details of search terms in each database

Database	Search term
PubMed	(glucosidase OR maltase OR sucrase OR amylase) AND (chalcone OR chalcones OR diphenylprop OR chalkone OR benzylideneacetophenone OR (phenyl styryl ketone))
Virtual Health Library	(glucosidase OR maltase OR sucrase OR amylase) AND (chalcone OR chalcones OR diphenylprop OR chalkone OR benzylideneacetophenone OR (phenyl styryl ketone))
Global Health Library	(glucosidase OR maltase OR sucrase OR amylase) AND (chalcone OR chalcones OR diphenylprop OR chalkone OR benzylideneacetophenone OR (phenyl styryl ketone))
Google Scholar	Search 1: glucosidase chalcone chalcones diphenylprop chalkone benzylideneacetophenone “phenyl styryl ketone” in the title of article Search 2: amylase chalcone chalcones diphenylprop chalkone benzylideneacetophenone “phenyl styryl ketone” in the title of article

**Figure 1:**
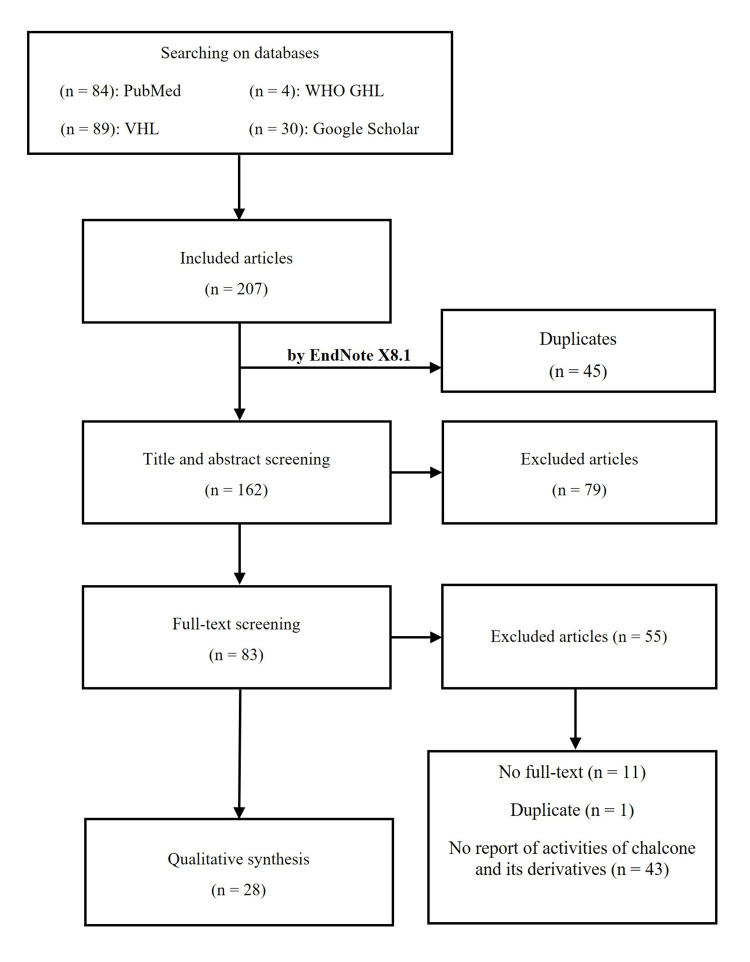
The PRISMA diagram of selected articles PRISMA: Preferred Reporting Items for Systematic Reviews and Meta-Analyses

In total, 207 articles were found in the four databases, including PubMed, Google Scholar, VHL, and GHL. After the selection step, 28 articles were included to extract data. The selection of studies is illustrated in Figure 1.

Many natural or synthetic chalcones have α-amylase and/or α-glucosidase inhibitory activities, including chalcones with a basic structural framework, azachalcones, bis-chalcones, chalcone analogs, chalcone oximes, coumarin-chalcone derivatives, cyclohexane chalcones, dihydrochalcones, and flavanone-coupled chalcones. Table 2 presents chalcones and their activities [3,5-31]. The structures of compounds are represented as SMILES notations (Simplified Molecular Input Line Entry System), which were obtained from PubChem (https://pubchem.ncbi.nlm.nih.gov/) by the name or chemical structure. Our set of compounds could be useful for future structure-activity relationship studies.

**Table 2:** The in vitro inhibitory activities against α-amylase and/or α-glucosidase of chalcones

Chalcones/source	Compounds	SMILES	α-amylase inhibitory activity	α-glucosidase inhibitory activity
Helal et al., (2022) [5]			IC_50_ (µM)	
Chalcone derivatives/ Dracaena cinnabari Balf. f. Resin	5	COC1=C(C=CC(=C1)O)CCC(=O)C2=CC=C(C=C2)	-	423.8
	6	C1=CC=C(C=C1)/C=C/C(=O)C2=C(C=C(C=C2)O)O	-	389.6
	7	O=C(C1=CC=C(O)C=C1OC)/C=C/C2=CC=C(O)C=C2	-	270.2
	Acarbose (PC)		-	47.3
Rocha et al., (2019) [3]			IC_50_ (µM) (mean ± SEM)^3^	
Chalcone derivatives (1-28) and chalcone analogues (29-41)/synthesized	4 (Butein)	C1=CC(=C(C=C1/C=C/C(=O)C2=C(C=C(C=C2)O)O)O)O	62 ± 4	21 ± 2
	24	C1=CC=C(C(=C1)C(=O)/C=C/C2=CC(=CC=C2)Cl)O	-	53 ± 1
	26	C1=CC=C(C(=C1)C(=O)/C=C/C2=C(C=C(C=C2)Cl)Cl)O	-	87 ± 3
	41	O=C(C1=CC=CC=C1O)/C=C/C=C/C2=CC=CC=C2[N+]([O-])=O	-	41 ± 1
	Acarbose (PC)		1.1 ± 0.2	357 ± 25
Dej-Adisai et al., (2021) [6]			IC_50_ (µg/mL)	
Chalcone derivatives /Bauhinia pulla	3,2’,4’-trihydroxy-4-methoxychalcone	COC1=CC=C(C=C1)/C=C/C(=O)C2=C(C=C(C=C2)O)O	-	1273.31
	4-methyl ether isoliquiritigenin	COC1=C(C=C(C=C1)/C=C/C(=O)C2=C(C=C(C=C2)O)O)O	-	220.05
	Acarbose (PC)		-	124.11
Cai et al., (2017) [10]			IC_50_ (µM) (mean ± SEM)^5^	
Chalcones (1a-1f, 2a-2f) and Bis-chalcones (1g-1m, 2g-2m)/ synthesized	1c	COC1=CC=C(C=C1)/C=C/C(=O)C2=C(C=C(C=C2)O)OC	-	58.7 ± 3.0
	1e	COC1=CC(=C(C=C1)/C=C/C(=O)C2=C(C=C(C=C2)OC)OC)OC	-	83.2 ± 1.0
	1f	COC1=C(C=C(C=C1)/C=C/C(=O)C2=CC(=C(C=C2)OC)OC)OC	-	123.1 ± 9.9
	1k	COC1=CC(=C(C=C1)/C=C/C(=O)C2=CC=C(C=C2)C(=O)/C=C/C3=C(C=C(C=C3)OC)OC)OC	-	66.9 ± 2.5
	2a	C1=CC(=CC=C1/C=C/C(=O)C2=C(C=C(C=C2O)O)O)O	-	71.1 ± 2.6
	2b	C1=CC(=CC=C1/C=C/C(=O)C2=CC(=C(C=C2)O)O)O	-	35.2 ± 0.2
	2c	C1=CC(=C(C=C1O)O)/C=C/C(=O)C2=C(C=C(C=C2)O)O	-	13.2 ± 2.7
	2d	C1=CC(=CC(=C1)O)/C=C/C(=O)C2=C(C=C(C=C2)O)O	-	42 ± 6.0
	2e	C1=CC(=C(C=C1O)O)/C=C/C(=O)C2=C(C=C(C=C2)O)O	-	12.5 ± 2.1
	2f	C1=CC(=C(C=C1/C=C/C(=O)C2=CC(=C(C=C2)O)O)O)O	-	15.6 ± 2.8
	2g	C1=CC(=CC(=C1)/C=C/C(=O)C2=C(C=C(C=C2)O)O)/C=C/C(=O)C3=C(C=C(C=C3)O)O	-	23.7 ± 0.3
	2h	C1=CC(=CC=C1/C=C/C(=O)C2=C(C=C(C=C2)O)O)/C=C/C(=O)C3=C(C=C(C=C3)O)O	-	22.5 ± 3.2
	2i	C1=CC(=CC=C1/C=C/C(=O)C2=CC(=C(C=C2)O)O)/C=C/C(=O)C3=CC(=C(C=C3)O)O	-	10.1 ± 1.7
	2j	C1=CC(=CC(=C1)C(=O)/C=C/C2=C(C=C(C=C2)O)O)C(=O)/C=C/C3=C(C=C(C=C3)O)O	-	5.5 ± 1.2
	2k	C1=CC(=CC=C1C(=O)/C=C/C2=C(C=C(C=C2)O)O)C(=O)/C=C/C3=C(C=C(C=C3)O)O	-	1.0 ± 0.1
	2l	C1=CC(=CC(=C1)C(=O)/C=C/C2=CC(=C(C=C2)O)O)C(=O)/C=C/C3=CC(=C(C=C3)O)O	-	6.5 ± 0.4
	2m	C1=CC(=CC(=C1)C(=O)/C=C/C2=CC=C(C=C2)O)C(=O)/C=C/C3=CC=C(C=C3)O	-	18.3 ± 0.7
	1-deoxynojirimycin (PC)		-	21.3 ± 8.7
Bale et al., (2018) [11]			IC_50_ (µM) (mean ± SEM)^3^	
Chalcones and Bis-chalcones derivatives/ synthesized	1	CC1=CC=C(C=C1)C(=O)/C=C/C2=CC=C(C=C2)OC	2.06 ± 0.04	-
	2	CC1=CC=C(C=C1)C(=O)/C=C/C2=CC=C(C=C2)Cl	1.85 ± 0.09	-
	3	CCOC1=C(C=C(C=C1)/C=C/C(=O)C2=CC=C(C=C2)C)OC	2.07 ± 0.08	-
	4	CC1=CC=C(C=C1)C(=O)/C=C/C2=CC=C(C=C2)SC	1.27 ± 0.7	-
	5	CC1=CC=C(C=C1)C(=O)/C=C/C2=CC=C(C=C2)OCC3=CC=CC=C3	1.99 ± 0.05	-
	6	CCOC1=CC=CC(=C1O)/C=C/C(=O)C2=CC=C(C=C2)C	2.26 ± 0.07	-
	7	COC1=CC=C(C=C1)/C=C/C(=O)C2=CC=C(C=C2)OC	1.92 ± 0.12	-
	8	COC1=CC=C(C=C1)C(=O)/C=C/C2=C(C=C(C=C2)Cl)Cl	1.97 ± 0.08	-
	9	COC1=CC=C(C=C1)C(=O)/C=C/C2=CC=C(C=C2)Br	1.98 ± 0.07	-
	10	COC1=CC=C(C=C1)C(=O)/C=C/C2=CC=C(C=C2)SC	1.25 ± 1.05	-
	11	COC1=CC=C(C=C1)C(=O)/C=C/C2=CC=C(C=C2)OCC3=CC=CC=C3	2.15 ± 0.07	-
	12	COC1=CC=C(C=C1)C(=O)/C=C/C2=CC=C(C=C2)C3=CC=CC=C3	1.99 ± 0.09	-
	13	CCOC1=CC=C(C=C1)/C=C/C(=O)C2=CC=C(C=C2)OC	2 ± 0.08	-
	14	C1=CC(=CC=C1/C=C/C(=O)/C=C/C2=CC=C(C=C2)Cl)Cl	1.72 ± 0.1	-
	15	C1=CC(=CC=C1/C=C/C(=O)/C=C/C2=CC=C(C=C2)Br)Br	1.8 ± 0.07	-
	16	COC1=CC=C(C=C1)/C=C/C(=O)/C=C/C2=CC=C(C=C2)OC	1.63 ± 0.18	-
	17	CSC1=CC=C(C=C1)/C=C/C(=O)/C=C/C2=CC=C(C=C2)SC	2.4 ± 0.09	-
	18	O=C(/C=C/C1=CC=CC(OCC)=C1O)/C=C/C2=CC=CC(OCC)=C2O	2.12 ± 0.1	-
	Acarbose (PC)		1.04 ± 0.3	-
Liu et al., (2014) [12]			IC_50_ (µM)	
Prenylated chalcone/ Humulus lupulus L.	Xanthohumol	CC(=CCC1=C(C(=C(C=C1O)OC)C(=O)/C=C/C2=CC=C(C=C2)O)O)C	-	8,8
	Acarbose (PC)		-	26,5
Mphahlele et al., (2021) [13]			IC_50_ (µM) (mean ± SD)^3^	
5-styryl-2-(4-tolylsulfonamido) chalcones/synthesized	2a	CC(=CCC1=C(C(=C(C=C1O)OC)C(=O)/C=C/C2=CC=C(C=C2)O)O)C	5.1 ± 0.3	4.2 ± 0.21
	2b	O=S(C1=CC=C(C)C=C1)(NC2=CC=C(/C=C/C3=CC=CC=C3)C=C2C(/C=C/C4=CC=CC(F)=C4)=O)=O	4.8 ± 0.25	5.4 ± 0.10
	2c	O=S(C1=CC=C(C)C=C1)(NC2=CC=C(/C=C/C3=CC=C(F)C=C3)C=C2C(/C=C/C4=CC=CC(F)=C4)=O)=O	13.6 ± 0.15	6.1 ± 0.56
	2d	O=S(C1=CC=C(C)C=C1)(NC2=CC=C(/C=C/C3=CC=C(Cl)C=C3)C=C2C(/C=C/C4=CC=CC(F)=C4)=O)=O	8.3 ± 0.4	8.1 ± 0.61
	2e	O=S(C1=CC=C(C)C=C1)(NC2=CC=C(/C=C/C3=CC=CC=C3)C=C2C(/C=C/C4=CC=C(F)C=C4)=O)=O	12.5 ± 0.18	12.5 ± 0.42
	2f	O=S(C1=CC=C(C)C=C1)(NC2=CC=C(/C=C/C3=CC=C(F)C=C3)C=C2C(/C=C/C4=CC=C(F)C=C4)=O)=O	7.6 ± 0.54	3.2 ± 0.33
	2g	O=S(C1=CC=C(C)C=C1)(NC2=CC=C(/C=C/C3=CC=C(Cl)C=C3)C=C2C(/C=C/C4=CC=C(F)C=C4)=O)=O	4.1 ± 0.61	9.7 ± 0.65
	2h	O=S(C1=CC=C(C)C=C1)(NC2=CC=C(/C=C/C3=CC=C(OC)C=C3)C=C2C(/C=C/C4=CC=C(F)C=C4)=O)=O	2.1 ± 0.53	5.9 ± 0.2
	α-Amylase inhibitor (PC)		0.39 ± 0.1	-
	Acarbose (PC)		-	0.95 ± 0.28
Jabeen et al., (2014) [14]			IC_50_ (µM)	
Chalcone derivatives/ synthesized	30	COC1=CC=CC(=C1)/C=C/C(=O)C2=CC=CC=C2	-	151.0
	34	C1=CC=C(C=C1)/C=C/C(=O)C2=CC=CC=C2O	-	15.0
	35	COC1=CC=C(C=C1)C(=O)/C=C/C2=CC=C(C=C2)N	-	20.2
	36	C1=CC=C(C(=C1)/C=C/C(=O)C2=CC=CC=C2O)O	-	49.4
	37	COC1=CC=CC(=C1)/C=C/C(=O)C2=CC=C(C=C2)N	-	93.0
	38	COC1=CC=CC(=C1O)C=CC(=O)C2=CC=CC=C2	-	130.0
	39	C1OC2=C(O1)C=C(C=C2)/C=C/C(=O)C3=C(C=C(C=C3))O	-	151.0
	40	C1OC2=C(O1)C=C(C=C2)C=CC(=O)C3=CC=CC=C4	-	1870.0
	41	C1=CC=C(C=C1)C(=O)/C=C/C2=CC=C(C=C2)[N+](=O)[O-]	-	3850.0
	Deoxynojirimycin (PC)		-	3.49
Sun et al., (2017) [7]			IC_50_ (µM) (SD less than ±10%)^3^	
Prenylchalconaringenins/Humulus lupulus L. Cannabaceae	3′-prenylchalconaringenin (1)	CC(=CCC1=C(C(=C(C=C1O)O)C(=O)/C=C/C2=CC=C(C=C2)O)O)C	85.92	22.42
	3′- geranylchalconaringenin (2)	CC(=CCC/C(=C/CC1=C(C(=C(C=C1O)O)C(=O)/C=C/C2=CC=C(C=C2)O)O)/C)C	20.46	1.08
	chalconaringenin (5)	C1=CC(=CC=C1/C=C/C(=O)C2=C(C=C(C=C2O)O)O)O	>100	20.02
	Acarbose (PC)		2.21	51.3
Seo et al., (2005) [15]			IC_50_ (µM)	
Aminochalcones (13-16) and sulfonamide chalcones (17-20)/ synthesized	15	C1=CC(=CC=C1/C=C/C(=O)C2=CC=C(C=C2)N)O	268.9	41
	16	C1=CC(=CC=C1C(=O)/C=C/C2=CC(=C(C=C2)O)O)N	NI	62.1
	17	O=S(C1=CC=C(C)C=C1)(NC2=CC=CC(C(/C=C/C3=CC=CC(O)=C3)=O)=C2)=O	37.3	12.4
	18	O=S(C1=CC=C(C)C=C1)(NC2=CC=CC(C(/C=C/C3=CC=C(O)C(O)=C3)=O)=C2)=O	16.8	15.6
	19	O=S(C1=CC=C(C)C=C1)(NC2=CC=C(C(/C=C/C3=CC=C(O)C=C3)=O)C=C2)=O	87.8	0.98
	20	O=S(C1=CC=C(C)C=C1)(NC2=CC=C(C(/C=C/C3=CC=C(O)C(O)=C3)=O)C=C2)=O	193.7	0.4
	Acarbose (PC)		-	60.8
Ansari et al., (2005) [16]			IC_50_ (µM) (mean ± SEM)	
Chalcones derivatives /synthesized	2	C1=CC=C(C=C1)C(=O)/C=C/C2=CC=CC=C2O	-	15 ± 0.14
	6	COC1=CC=CC(=C1)/C=C/C(=O)C2=CC=CC=C2	-	151 ± 1.4
	13	COC1=CC=CC(=C1O)/C=C/C(=O)C2=CC=CC=C2	-	130 ± 2.91
	17	C1OC2=C(O1)C=C(C=C2)/C=C/C(=O)C3=CC=CC=C3	-	187 ± 8.4
	18	C1=CC=C(C(=C1)/C=C/C(=O)C2=CC=CC=C2O)O	-	46.4 ± 2.9
	19	C1OC2=C(O1)C=C(C=C2)/C=C/C(=O)C3=CC=C(C=C3)N	-	93 ± 0.73
	21	COC1=CC=C(C=C1)/C=C/C(=O)C2=CC=C(C=C2)N	-	20.2 ± 1.42
	22	COC1=C(C=C(C=C1)/C=C/C(=O)C2=CC=CC=C2O)OC	-	151 ± 0.05
	23	C1=CC=C(C=C1)C(=O)/C=C/C2=CC=C(C=C2)[N+](=O)[O-]	-	385 ± 5.60
	Deoxynojirimycin (PC)		-	425 ± 8.14
Lin et al., (2020) [17]			Inhibition ratio (%)	
Chalcone derivatives/ synthesized	1	O=C(C1=CC=CC=C1OCCBr)/C=C/C2=CC=CC=C2	-	9.2 (10 mmol/L) 15.75 (20 mmol/L)
	2	O=C(C1=CC=CC=C1OCC(Br)C)/C=C/C2=CC=CC=C2	-	5.28 (10 mmol/L) 10.44 (20 mmol/L)
	3	O=C(C1=CC=CC=C1OCC(Br)CC)/C=C/C2=CC=CC=C2	-	NI (10 mmol/L) 2.11 (20 mmol/L)
	Acarbose (PC)		-	38.33 (10 mmol/L) 49.32 (20 mmol/L)
Tang et al., (2012) [18]			IC_50_ (µM) (mean ± SEM)^3^	
Oleanolic acid derivatives chalcones/ synthesized	1b	O=C([C@]1(CC[C@@]2(C)[C@]3(C)CC[C@@]4([H])C5(C)C)CCC(C)(C)C[C@@]1(C)C2=CCC3[C@@]4(C)CCC5=O)OC6=CC=C(C(/C=C/C7=CC=C(OC)C=C7)=O)C=C6	-	219.8 ± 5.3
	2a	O=C([C@@]12CC[C@@]3(C)[C@]4(C)CC[C@@]5([H])C(C)(C)C(NC6=C7C=CC=C6)=C7C[C@]5(C)C4CC=C3[C@]1(C)CC(C)(C)CC2)OC8=CC=C(C(/C=C/C9=CC=C(Br)C=C9)=O)C=C8	-	217.2 ± 6.7
	2b	O=C([C@@]12CC[C@@]3(C)[C@]4(C)CC[C@@]5([H])C(C)(C)C(NC6=C7C=CC=C6)=C7C[C@]5(C)C4CC=C3[C@]1(C)CC(C)(C)CC2)OC8=CC=C(C(/C=C/C9=CC=CO9)=O)C=C8	-	47.5 ± 4.1
	3	O=C([C@]1(CC[C@@]2(C)[C@]3(C)CC[C@@]4([H])C5(C)C)CCC(C)(C)C[C@@]1(C)C2=CCC3[C@@]4(C)C=CC5=O)OC6=CC=C(C(/C=C/C7=CC=CC=C7)=O)C=C6	-	272.1 ± 13.2
	Oleanolic acid		-	102.3 ± 2.4
	Acarbose		-	362.3 ± 7.6
Sukanadi et al., (2005) [20]			IC_50_ (µM) (mean ± SD)^3^	
Sulfonamide chalcones/synthesized	3a	O=S(C1=CC=C(C)C=C1)(NC2=CC=CC=C2C(/C=C/C3=CC=C(OC)C(OC)=C3)=O)=O	-	76.7 ± 5.99
	3b	O=S(C1=CC=C(C)C=C1)(NC2=CC=CC(C(/C=C/C3=CC=C(OC)C(OC)=C3)=O)=C2)=O	-	30.2 ± 4.4
	3c	O=S(C1=CC=C(C)C=C1)(NC2=CC=C(C(/C=C/C3=CC=C(OC)C(OC)=C3)=O)C=C2)=O		1.04 ± 0.19
	3d	O=S(C1=CC=C(C)C=C1)(NC2=CC=C(C(/C=C/C3=CC=CC=C3OC)=O)C=C2)=O		28.6 ± 4.3
	3e	O=S(C1=CC=C(C)C=C1)(NC2=CC=C(C(/C=C/C3=CC=CC(OC)=C3)=O)C=C2)=O		4.82 ± 1.08
	3f	O=S(C1=CC=C(C)C=C1)(NC2=CC=C(C(/C=C/C3=CC=C(OC)C=C3)=O)C=C2)=O		10.5 ± 0.67
	3g	O=S(C1=CC=C(C)C=C1)(NC2=CC=C(C(/C=C/C3=CC=C(F)C=C3)=O)C=C2)=O		15.5 ± 0.85
	3h	O=S(C1=CC=C(C)C=C1)(NC2=CC=C(C(/C=C/C3=CC=C(Cl)C=C3)=O)C=C2)=O		14.6 ± 0.76
	3i	O=S(C1=CC=C(C)C=C1)(NC2=CC=C(C(/C=C/C3=CC=C(Br)C=C3)=O)C=C2)=O		18.9 ± 0.46
	3j	O=S(C1=CC=C(C)C=C1)(NC2=CC=C(C(/C=C/C3=CC=C([N+]([O-])=O)C=C3)=O)C=C2)=O		80.3 ± 2.59
	3l	O=S(C1=CC=C(C(/C=C/C2=CC=C(OC)C(OC)=C2)=O)C=C1)(NC3=CC=C(F)C=C3)=O		135.9 ± 11.06
	Acarbose (PC)		-	93.6 ± 0.49
Sun et al., (2015) [21]			IC_50_ (µM) (SD less than ±10%)^2^	
Chalcone derivatives/ synthesized	1	CC(=CCC1=CC(=C(C=C1O)O)C(=O)/C=C/C2=CC=C(C=C2)O)C	-	31.31
	14	CC(=CCC/C(=C/CC1=CC(=C(C=C1O)O)C(=O)/C=C/C2=CC=C(C=C2)O)/C)C	-	16.31
	15	CC(=CCC1=CC(=C(C(=C1O)CC=C(C)C)O)C(=O)/C=C/C2=CC=C(C=C2)O)C	-	6.9
	16	CC(=CCC/C(=C/CC1=CC(=C(C(=C1O)C/C=C(\C)/CCC=C(C)C)O)C(=O)/C=C/C2=CC=C(C=C2)O)/C)C	-	0.9
	17	CC(=CCC/C(=C/CC1=CC(=C(C(=C1O)CC=C(C)C)O)C(=O)/C=C/C2=CC=C(C=C2)O)/C)C	-	11.51
	18	CC(=CCC/C(=C/CC1=C(C(=CC(=C1O)C(=O)/C=C/C2=CC=C(C=C2)O)CC=C(C)C)O)/C)C	-	9
	Acarbose (PC)		-	51.32
Chinthala et al., (2015) [22]			IC_50_ (µM)	
chalcone triazoles/ synthesized	4h	CC1(CCC2=C(O1)C=CC(=C2O)C(=O)/C=C/C3=CC=C(C=C3)OCC4=CN(N=N4)C5=CC=C(C=C5)Cl)C	-	127.90
	4m	CCC1=CC=CC=C1N2C=C(N=N2)COC3=CC=C(C=C3)/C=C/C(=O)C4=C(C5=C(C=C4)OC(CC5)(C)C)O	-	67.77
	4p	CCCCCN1C=C(N=N1)COC2=CC=C(C=C2)/C=C/C(=O)C3=C(C=C4C(=C3)CCC(O4)(C)C)O	-	74.94
	4s	CCCCCN1C=C(N=N1)COC2=CC=C(C=C2)/C=C/C(=O)C3=C(C4=C(C=C3)OC(CC4)(C)C)O	-	102.1
	Acarbose (PC)			23.87
Mphahlele et al., (2021) [23]			IC_50_ (µM) (mean ± SD)^3^	
Chalcone derivatives/ synthesized	3a	O=C(C1=CC(/C=C/C2=CC=CC=C2)=CC=C1N)/C=C/C3=CC=CC(F)=C3	10.7 ± 0.21	17.8 ± 0.32
	3b	O=C(C1=CC(/C=C/C2=CC=C(F)C=C2)=CC=C1N)/C=C/C3=CC=CC(F)=C3	15.6 ± 0.6	6.1 ± 0.24
	3c	O=C(C1=CC(/C=C/C2=CC=C(Cl)C=C2)=CC=C1N)/C=C/C3=CC=CC(F)=C3	2.4 ± 0.1	12.6 ± 0.31
	3d	O=C(C1=CC(/C=C/C2=CC=C(OC)C=C2)=CC=C1N)/C=C/C3=CC=CC(F)=C3	7 ± 0.45	9.4 ± 0.5
	3e	O=C(C1=CC(/C=C/C2=CC=CC=C2)=CC=C1N)/C=C/C3=CC=C(F)C=C3	1.6 ± 0.52	5.1 ± 0.61
	3f	O=C(C1=CC(/C=C/C2=CC=C(F)C=C2)=CC=C1N)/C=C/C3=CC=C(F)C=C3	9.5 ± 0.41	6.9 ± 0.37
	3g	O=C(C1=CC(/C=C/C2=CC=C(Cl)C=C2)=CC=C1N)/C=C/C3=CC=C(F)C=C3	1.7 ± 0.25	19.2 ± 0.47
	3h	O=C(C1=CC(/C=C/C2=CC=C(OC)C=C2)=CC=C1N)/C=C/C3=CC=C(F)C=C3	7.6 ± 0.2	10.5 ± 0.18
	Acarbose (PC)		1.03 ± 0.05	0.95 ± 0.28
	α-Amylase inhibitor (PC)		0.31 ± 0.05	-
He et al., (2021) [8]			IC_50_ (µM) (mean ± SD)^3^	
Chalcone derivatives /Alpinia katsumadai	Katsumadainol A_1_	O=C(C1=C(OC)C=C(O)C=C1O)[C@H]2[C@H](C3=CC=C(O)C=C3)O[C@@H](CCC4=CC=C(O)C=C4)C[C@H]2/C=C/C5=CC=C(O)C=C5	-	9.4 ± 0.3
	Katsumadainol A_2_	O=C(C1=C(OC)C=C(O)C=C1O)[C@H]2[C@H](C3=CC=C(O)C=C3)O[C@H](CCC4=CC=C(O)C=C4)C[C@H]2/C=C/C5=CC=C(O)C=C5	-	7.2 ± 0.2
	Katsumadainol A_3_	O=C(C1=C(OC)C=C(O)C([C@H]2[C@@H](O)[C@@H](C3=CC=C(O)C=C3)O[C@@H](CCC4=CC=C(O)C=C4)C2)=C1O)/C=C/C5=CC=C(O)C=C5	-	22 ± 1.6
	Katsumadainol A_5_	O=C(C1=C(OC)C=C(O)C([C@H]2[C@H](O)[C@@H](C3=CC=C(O)C=C3)O[C@H](CCC4=CC=C(O)C=C4)C2)=C1O)/C=C/C5=CC=C(O)C=C5	-	17.3 ± 3.2
	Katsumadainol A_6_	O=C(C1=C(OC)C=C(O)C([C@@H]2[C@H]([C@@H](C3=CC=C(O)C=C3)/C=C/C[C@@H](O)CCC4=CC=C(O)C=C4)[C@H](C5=CC=C(O)C=C5)O[C@H](CCC6=CC=C(O)C=C6)C2)=C1O)/C=C/C7=CC=C(O)C=C7	-	20.6 ± 4.1
	Katsumadainol A_7_	O=C(C1=C(OC)C=C(O)C([C@@H]2[C@H]([C@@H](C3=CC=C(O)C=C3)/C=C/C[C@@H](O)CCC4=CC=C(O)C=C4)[C@H](C5=CC=C(O)C=C5)O[C@H](CCC6=CC=C(O)C=C6)C2)=C1O)/C=C/C7=CC=C(O)C=C7	-	23.8 ± 5.4
	Katsumadainol A_10_	O=C(C1=C2C([C@](C[C@H](C3=CC=C(O)C=C3)O2)([H])C[C@@H](CCC4=CC=C(O)C=C4)O5)=C5C=C1OC)/C=C/C6=CC=C(O)C=C6	-	22.5 ± 1.8
	Katsumadainol A_11_	CC(O[C@@H](C/C=C/[C@@H](C1=C(O)C=C(OC)C(C(/C=C/C2=CC=C(O)C=C2)=O)=C1O)C3=CC=C(O)C=C3)CCC4=CC=C(O)C=C4)=O	-	3.1 ± 0.2
	Katsumadainol A_12_	CC(O[C@@H](C/C=C/[C@H](C1=C(O)C=C(OC)C(C(/C=C/C2=CC=C(O)C=C2)=O)=C1O)C3=CC=C(O)C=C3)CCC4=CC=C(O)C=C4)=O	-	5.8 ± 0
	Katsumadainol A_13_	O=C(C1=C(OC)C(OC)=C(O)C([C@@H](C2=CC=C(O)C=C2)/C=C/C[C@@H](O)CCC3=CC=C(O)C=C3)=C1O)/C=C/C4=CC=C(O)C=C4	-	6.2 ± 0.2
	Katsumadainol A_14_	O=C(C1=C(OC)C(OC)=C(O)C([C@H](C2=CC=C(O)C=C2)/C=C/C[C@@H](O)CCC3=CC=C(O)C=C3)=C1O)/C=C/C4=CC=C(O)C=C4	-	11.2 ± 1.6
	Katsumadainol A_15_	O=C(C1=C(OC)C=C(O)C([C@@H](C2=CC=C(O)C=C2)/C=C/C[C@@H](O)CCC3=CC=C(O)C(OC)=C3)=C1O)/C=C/C4=CC=C(O)C=C4	-	11.5 ± 0.6
	Katsumadainol A_16_	O=C(C1=C(OC)C=C(O)C([C@H](C2=CC=C(O)C=C2)/C=C/C[C@@H](O)CCC3=CC=C(O)C(OC)=C3)=C1O)/C=C/C4=CC=C(O)C=C4	-	7.6 ± 0
	calyxin F (17)	COC1=C(C(=C(C(=C1)O)[C@@H]2C[C@@H](O[C@H](C2)C3=CC=C(C=C3)O)CCC4=CC=C(C=C4)O)O)C(=O)/C=C/C5=CC=C(C=C5)O		5.4 ± 0.1
	epicalyxin F (18)	COC1=C(C(=C(C(=C1)O)[C@@H]2C[C@@H](O[C@@H](C2)C3=CC=C(C=C3)O)CCC4=CC=C(C=C4)O)O)C(=O)/C=C/C5=CC=C(C=C5)O		2.9 ± 0.1
	(3S,5S,6S,7R)-6-hydroxycalyxin F (19)	COC1=C(C(=C(C(=C1)O)[C@@H]2C[C@@H](O[C@@H]([C@H]2O)C3=CC=C(C=C3)O)CCC4=CC=C(C=C4)O)O)C(=O)/C=C/C5=CC=C(C=C5)O		29.5 ± 3.8
	(3S,5S,6S,7S)-6-hydroxycalyxin F (20)	COC1=C(C(=C(C(=C1)O)[C@@H]2C[C@@H](O[C@H]([C@H]2O)C3=CC=C(C=C3)O)CCC4=CC=C(C=C4)O)O)C(=O)/C=C/C5=CC=C(C=C5)O		10.1 ± 0.6
	calyxin L (21)	COC1=C(C2=C([C@@H](C[C@@H](O2)C3=CC=C(C=C3)O)C[C@H](CCC4=CC=C(C=C4)O)O)C(=C1)O)C(=O)/C=C/C5=CC=C(C=C5)O		4.3 ± 0.2
	epicalyxin B (22)	COC1=C(C(=C(C(=C1)O)[C@H](/C=C/C[C@H](CCC2=CC=C(C=C2)O)O)C3=CC=C(C=C3)O)O)C(=O)/C=C/C4=CC=C(C=C4)O		16.6 ± 1.7
	calyxin B (23)	COC1=C(C(=C(C(=C1)O)[C@@H](/C=C/C[C@H](CCC2=CC=C(C=C2)O)O)C3=CC=C(C=C3)O)O)C(=O)/C=C/C4=CC=C(C=C4)O		13.1 ± 0.3
	alpinnanin A (24)	COC1=C(C(=C(C(=C1)O)[C@@H](/C=C/C[C@@H](CCC2=CC=CC=C2)O)C3=CC=C(C=C3)O)O)C(=O)/C=C/C4=CC=CC=C4		11.4 ± 1.8
	alpinnanin B (25)	COC1=C(C(=C(C(=C1)O)[C@@H](/C=C/C[C@H](CCC2=CC=CC=C2)O)C3=CC=C(C=C3)O)O)C(=O)/C=C/C4=CC=CC=C4		19.4 ± 1.6
	calyxin H (26)	COC1=C(C(=C(C(=C1)O)[C@@H](/C=C/C[C@H](CCC2=CC=CC=C2)O)C3=CC=C(C=C3)O)O)C(=O)/C=C/C4=CC=C(C=C4)O		8.9 ± 1.7
	epi-calyxin H (27)	COC1=C(C(=C(C(=C1)O)[C@H](/C=C/C[C@H](CCC2=CC=CC=C2)O)C3=CC=C(C=C3)O)O)C(=O)/C=C/C4=CC=C(C=C4)O		6 ± 0.2
	katsumain C (28)	COC1=C(C(=C(C(=C1)O)[C@H](/C=C/C[C@@H](CCC2=CC=C(C=C2)O)O)C3=CC=C(C=C3)O)O)C(=O)/C=C/C4=CC=CC=C4		5.4 ± 2
	7-epi-katsumain C (29)	COC1=C(C(=C(C(=C1)O)[C@@H](/C=C/C[C@@H](CCC2=CC=C(C=C2)O)O)C3=CC=C(C=C3)O)O)C(=O)/C=C/C4=CC=CC=C4		6.8 ± 1.5
	Acarbose		-	170.9 ± 3.2
Saleem et al., (2021) [25]			IC_50_ (µM) (mean ± SEM)	
Azachalcones/ synthesized	3	COC1=CC=CC=C1/C=C/C(=O)C2=CC=CC=N2	23.08 ± 0.03	26.08 ± 0.43
	4	COC1=CC(=CC(=C1)/C=C/C(=O)C2=CC=CC=N2)OC	61.01 ± 0.17	64.57 ± 0.07
	5	COC1=C(C(=C(C=C1)/C=C/C(=O)C2=CC=CC=N2)OC)OC	24.57 ± 0.07	27.57 ± 0.07
	6	O=C(C1=NC=CC=C1)/C=C/C2=CC=C(OC)C=C2F	24.94 ± 0.12	27.13 ± 0.08
	7	O=C(C1=NC=CC=C1)/C=C/C2=CC=C(F)C(OC)=C2	45.04 ± 0.52	47.71 ± 0.16
	8	O=C(C1=NC=CC=C1)/C=C/C2=CC=CC(OC)=C2Cl	37.91 ± 0.18	39.02 ± 0.11
	9	O=C(C1=NC=CC=C1)/C=C/C2=C(OC)C=CC=C2Br	69.71 ± 0.16	68.33 ± 0.12
	10	O=C(C1=NC=CC=C1)/C=C/C2=CC(OC)=C(Br)C(OC)=C2	47.91 ± 0.18	49.02 ± 0.11
	11	O=C(C1=NC=CC=C1)/C=C/C2=CC(OC)=C(OC)C=C2Br	63.08 ± 0.03	64.94 ± 0.12
	12	O=C(C1=NC=CC=C1)/C=C/C2=CC=C(Cl)C=C2	45.04 ± 0.52	47.7 ± 0.16
	13	O=C(C1=NC=CC=C1)/C=C/C2=CC=C(Cl)C=C2Cl	34.94 ± 0.12	37.13 ± 0.08
	14	O=C(C1=NC=CC=C1)/C=C/C2=CC(Cl)=CC=C2O	40.04 ± 0.2	41.13 ± 0.18
	15	O=C(C1=NC=CC=C1)/C=C/C2=CC(Cl)=CC(Cl)=C2O	88.15 ± 0.12	87.13 ± 0.12
	16	O=C(C1=NC=CC=C1)/C=C/C2=CC=CC([N+]([O-])=O)=C2	27.57 ± 0.07	29.13 ± 0.18
	17	O=C(C1=NC=CC=C1)/C=C/C2=CC=C([N+]([O-])=O)C=C2	77.33 ± 0.02	79.71 ± 0.16
	18	O=C(C1=NC=CC=C1)/C=C/C2=CC([N+]([O-])=O)=CC=C2Cl	51.94 ± 0.12	53.33 ± 0.02
	19	O=C(C1=NC=CC=C1)/C=C/C2=CC=C3C=CC=CC3=C2	77.27 ± 0.18	75.04 ± 0.52
	21	O=C(C1=CC=CN=C1)/C=C/C2=CC(OC)=CC(OC)=C2	86.33 ± 0.02	89.01 ± 0.12
	22	O=C(C1=CC=CN=C1)/C=C/C2=CC=C(OC)C(OC)=C2OC	89.71 ± 0.16	85.97 ± 0.19
	23	O=C(C1=CC=CN=C1)/C=C/C2=CC=C(OC)C=C2F	43.08 ± 0.03	46.08 ± 0.43
	24	O=C(C1=CC=CN=C1)/C=C/C2=CC=C(F)C(OC)=C2	87.27 ± 0.18	89.71 ± 0.16
	25	O=C(C1=CC=CN=C1)/C=C/C2=CC=CC(OC)=C2Cl	81.94 ± 0.12	82.13 ± 0.08
	26	O=C(C1=CC=CN=C1)/C=C/C2=CC(OC)=C(Br)C(OC)=C2	85.33 ± 0.02	87.47 ± 0.13
	28	O=C(C1=CC=CN=C1)/C=C/C2=CC([N+]([O-])=O)=CC=C2Cl	26.9 ± 0.12	27.99 ± 0.09
	Acarbose (PC)		18.08 ± 0.07	18.67 ± 0.09
Ali et al., (2021) [24]			IC_50_ (µM) (mean ± SEM)	
Chalcone derivatives/ synthesized	1	O=C(C1=CC=C2C=CC=CC2=C1)/C=C/C3=CC=CC(OC)=C3O	2.38 ± 0.248	-
	2	O=C(C1=CC=C2C=CC=CC2=C1)/C=C/C3=CC(OC)=C(OC)C(OC)=C3	1.62 ± 0.321	-
	3	O=C(C1=CC=C2C=CC=CC2=C1)/C=C/C3=CC=CC(OC)=C3	1.55 ± 0.051	-
	4	O=C(C1=CC=C2C=CC=CC2=C1)/C=C/C3=CC=C(N(C)C)C=C3	1.47 ± 0.481	-
	5	O=C(C1=CC=C2C=CC=CC2=C1)/C=C/C3=CC=CC(O)=C3	2.44 ± 0.274	-
	6	O=C(C1=CC=C2C=CC=CC2=C1)/C=C/C3=CC=C(O)C(O)=C3	1.71 ± 0.216	-
	7	O=C(C1=CC=C2C=CC=CC2=C1)/C=C/C3=CC=C(Cl)C=C3	2.48 ± 0.139	-
	8	O=C(C1=CC=C2C=CC=CC2=C1)/C=C/C3=CC=C([N+]([O-])=O)C=C3	2.15 ± 0.097	-
	9	O=C(C1=CC=C2C=CC=CC2=C1)/C=C/C3=CC=C(C)C=C3	1.67 ± 0.284	-
	10	O=C(C1=CC=C2C=CC=CC2=C1)/C=C/C3=CC(Br)=CC=C3OC	2.57 ± 0.252	-
	11	O=C(C1=CC=C2C=CC=CC2=C1)/C=C/C3=CC=C(F)C=C3OC	2.81 ± 0.16	-
	12	O=C(C1=CC=C2C=CC=CC2=C1)/C=C/C3=CC=C(OC)C(OC)=C3	1.53 ± 0.208	-
	13	O=C(C1=CC=C2C=CC=CC2=C1)/C=C/C3=CC=CC(OC)=C3OC	1.87 ± 0.039	-
	14	O=C(C1=CC=C2C=CC=CC2=C1)/C=C/C3=CC=C(OC)C=C3OC	1.62 ± 0.039	-
	15	O=C(C1=CC=C2C=CC=CC2=C1)/C=C/C3=C(OC)C=CC=C3OC	1.88 ± 0.027	-
	16	O=C(C1=CC=C2C=CC=CC2=C1)/C=C/C3=CC=C(O)C=C3O	2.52 ± 0.044	-
	Acarbose		1.34 ± 0.326	-
Bashary et al., (2019) [31]			IC_50_ (μg/mL)	
Dihydrochalcones/ synthesized	3a	C1=CC=C(C=C1)C(CC(=O)C2=CC=CC=C2)NC3=CC=CC=C3	25.7	-
	3b	CC1=CC=C(C=C1)NC(CC(=O)C2=CC=CC=C2)C3=CC=CC=C3	225.89	-
	3c	COC1=CC=C(C=C1)C(CC(=O)C2=CC=CC=C2)NC3=CC=CC=C3	177.82	-
	3d	COC1=CC=C(C=C1)NC(CC(=O)C2=CC=CC=C2)C3=CC=CC=C4	251.18	-
	3e	C1=CC=C(C=C1)C(CC(=O)C2=CC=CC=C2)NC3=CC=C(C=C3)Cl	23.18	-
	3f	C1=CC=C(C=C1)C(CC(=O)C2=CC=CC=C2)NC3=CC=C(C=C3)Br	48.17	-
	Acarbose		891.25	-
Fandaklı et al., (2018) [26]			IC_50_ (µM) (mean ± SD)	
Chalcone oximes/ synthesized	2a	OC1=CC(C(/C=C/C2=CC=CC=C2)=N/O)=CC=C1	-	1.61 ± 0.16
	2b	OC1=CC=C(C(/C=C/C2=CC=CC=C2)=N/O)C=C1	-	3.36 ± 0.58
	2c	COC1=CC=CC=C1/C=C/C(C2=CC=CC=C2OC)=N\O	-	11.2 ± 2.04
	2d	COC1=CC=CC=C1C(/C=C/C2=CC=CC(OC)=C2OC)=N/O	-	9.2 ± 2.9
	2e	COC1=CC=C(/C=C/C(C2=CC=CC=C2OC)=N\O)C(OC)=C1OC	-	9.39 ± 0.94
	2f	COC1=CC=C(C(/C=C/C2=CC=CC=C2OC)=N/O)C=C1	-	12.5 ± 0.98
	2g	COC1=CC=C(C(/C=C/C2=CC=CC(OC)=C2)=N/O)C=C1	-	14 ± 2
	2h	COC1=CC=C(C(/C=C/C2=CC=CC(OC)=C2OC)=N/O)C=C1	-	25.5 ± 0.38
	Acarbose		-	13.3 ± 1.26
Hu et al., (2022) [27]			IC_50_ (µM) (mean ± SEM)	
Coumarin-chalcone derivatives/ synthesized	3a	C1=CC=C(C=C1)/C=C/C(=O)C2=CC3=CC=CC=C3OC2=O	-	125.26 ± 1.18
	3b	CC1=CC=C(C=C1)/C=C/C(=O)C2=CC3=CC=CC=C3OC2=O	-	95.23 ± 1.35
	3c	COC1=CC=CC=C1/C=C/C(=O)C2=CC3=CC=CC=C3OC2=O	-	60.89 ± 2.74
	3d	COC1=CC=CC(=C1)/C=C/C(=O)C2=CC3=CC=CC=C3OC2=O	-	96.39 ± 1.37
	3e	COC1=CC=C(C=C1)/C=C/C(=O)C2=CC3=CC=CC=C3OC2=O	-	105.18 ± 1.98
	3f	CSC1=CC=C(C=C1)/C=C/C(=O)C2=CC3=CC=CC=C3OC2=O	-	75.53 ± 0.98
	3g	O=C1C(C(/C=C/C2=CC=CC=C2F)=O)=CC3=C(O1)C=CC=C3	-	48.36 ± 1.42
	3h	C1=CC=C2C(=C1)C=C(C(=O)O2)C(=O)/C=C/C3=CC(=CC=C3)F	-	45.68 ± 1.28
	3i	C1=CC=C2C(=C1)C=C(C(=O)O2)C(=O)/C=C/C3=CC=C(C=C3)F	-	35.68 ± 0.28
	3j	O=C1C(C(/C=C/C2=CC=C(F)C=C2F)=O)=CC3=C(O1)C=CC=C3	-	30.3 ± 2.53
	3k	C1=CC=C2C(=C1)C=C(C(=O)O2)C(=O)/C=C/C3=CC(=C(C=C3)F)F	-	49.68 ± 3.28
	3l	C1=CC=C2C(=C1)C=C(C(=O)O2)C(=O)/C=C/C3=CC=CC=C3C(F)(F)F	-	71.52 ± 2.14
	3m	C1=CC=C2C(=C1)C=C(C(=O)O2)C(=O)/C=C/C3=CC(=CC=C3)C(F)(F)F	-	64.71 ± 1.82
	3n	C1=CC=C2C(=C1)C=C(C(=O)O2)C(=O)/C=C/C3=CC=C(C=C3)C(F)(F)F	-	53.58 ± 1.95
	3o	C1=CC=C2C(=C1)C=C(C(=O)O2)C(=O)/C=C/C3=CC=CC=C3Cl	-	59.68 ± 1.73
	3p	C1=CC=C2C(=C1)C=C(C(=O)O2)C(=O)/C=C/C3=CC(=CC=C3)Cl	-	52.62 ± 2.45
	3q	C1=CC=C2C(=C1)C=C(C(=O)O2)C(=O)/C=C/C3=CC=C(C=C3)Cl	-	29.74 ± 2.68
	3r	C1=CC=C2C(=C1)C=C(C(=O)O2)C(=O)/C=C/C3=CC=CC=C3Br	-	38.56 ± 1.87
	3s	C1=CC=C2C(=C1)C=C(C(=O)O2)C(=O)/C=C/C3=CC(=CC=C3)Br	-	35.56 ± 2.18
	3t	C1=CC=C2C(=C1)C=C(C(=O)O2)C(=O)/C=C/C3=CC=C(C=C3)Br	-	24.09 ± 2.36
	3u	C1=CC=C2C(=C1)C=C(C(=O)O2)C(=O)/C=C/C3=CC=CS3	-	109.23 ± 2.69
	3v	O=C1C(C(/C=C/C(N2)=CC3=C2C=CC=C3)=O)=CC4=C(O1)C=CC=C4	-	103.31 ± 1.45
	Acarbose		-	259.9 ± 1.06
Chatsumpun et al., (2017) [28]			IC_50_ (µM) (mean ± SEM)	
Cyclohexane chalcones and flavanone-coupled chalcones/ Boesenbergia rotunda	3	CC1=CC[C@H]([C@@H]([C@@H]1CC=C(C)C)C(=O)C2=C(C=C(C=C2O)OC)O)C3=CC=CC=C3	-	12.7 ± 1.3
	4	CC1=CC[C@H]([C@@H]([C@@H]1CC=C(C)C)C(=O)C2=C(C=C(C=C2OC)O)O)C3=CC=CC=C3	-	7.5 ± 0.6
	7	CC1=CC[C@H]([C@@H]([C@@H]1CC=C(C)C)C(=O)C2=C(C=C(C=C2O)O)O)C3=CC=CC=C3	-	4.6 ± 0.4
	9	O=C1CC(C2=CC=CC=C2)OC3=C1C(O)=C(C(C4=CC=CC=C4)CC(C5=C(O)C=C(OC)C=C5O)=O)C(O)=C3	-	2.4 ± 0.4
	10	C1=CC=C(C=C1)CCC(=O)C2=C(C=C(C=C2O)O)O	-	32.0 ± 2.2
	12	O=C1CC(C2=CC=CC=C2)OC3=C1C(O)=C(C(C4=CC=CC=C4)CC(C5=C(O)C=C(O)C=C5OC)=O)C(O)=C3	-	3.4 ± 0.9
	13	O=C1CC(C2=CC=CC=C2)OC3=C1C(O)=C(C(C4=CC=CC=C4)CC(C5=C(O)C=C(O)C=C5O)=O)C(O)=C3	-	1.3 ± 0.2
	Acarbose			1155.5 ± 23
Helal et al., (2021) [29]			IC_50_ (µg/mL)	
Chalcone-dihydrochalcone dimer/Dracaena cinnabari	Dracidione	OC1=C(CC2=C(O)C=C(OC)C(CCC(C3=CC=CC=C3)=O)=C2)C(O)=CC=C1C(/C=C/C4=CC=CC=C4)=O	-	40.2
	Acarbose		-	30.5
Tanaka et al., (2005) [30]			IC_50_ (µg/mL)	
Dihydrochalcone glucosides/Balanophora tobiracola	5	O=C(O[C@@H]1[C@@H](COC(C2=CC(O)=C(O)C(O)=C2)=O)O[C@@H](OC3=CC(O)=C(C(CCC4=CC=C(O)C(O)=C4)=O)C(O)=C3)[C@H](O)C1O)C5=CC(O)=C(O)C(O)=C5	-	1.8
	6	O=C(C1=CC(O)=C(C(O)=C1C2=C(O)C(O)=C(O)C=C23)O)O[C@@H]4[C@H](O[C@H]([C@@H](C4O)O)OC5=CC(O)=C(C(O)=C5)C(CCC6=CC=C(C(O)=C6)O)=O)COC3=O	-	1.6
	7	O=C(C1=CC(O)=C(C(O)=C1C2=C(O)C(O)=C(O)C=C23)O)O[C@@H]4[C@H](O[C@H]([C@@H](C4OC(C5=CC(O)=C(O)C(O)=C5)=O)O)OC6=CC(O)=C(C(O)=C6)C(CCC7=CC=C(C(O)=C7)O)=O)COC3=O	-	0.4
	8	O=C(C1=CC(O)=C(C(O)=C1C2=C(O)C(O)=C(O)C=C23)O)O[C@@H]4[C@H](O[C@H]([C@@H](C4OC(/C=C/C5=CC=C(O)C(O)=C5)=O)O)OC6=CC(O)=C(C(O)=C6)C(CCC7=CC=C(C(O)=C7)O)=O)COC3=O	-	1.1
	9	O=C(O[C@@H]1[C@@H](CO2)O[C@@H](OC3=CC(O)=C(C(CCC4=CC=C(O)C=C4)=O)C(O)=C3)[C@H](O)C1OC(C5=CC(O)=C(C(O)=C5)O)=O)C6=CC(O)=C(O)C(O)=C6C7=C(C(O)=C(C=C7C2=O)O)O	-	0.8
Tang et al., (2014) [19]			IC_50_ (µM) (mean ± SD)	
Oleanolic acid derivatives chalcones/ synthesized	1a	C[C@]12CCC(=O)C([C@@H]1CC[C@@]3([C@@H]2CC=C4[C@]3(CC[C@@]5([C@H]4CC(CC5)(C)C)C(=O)OC6=CC=C(C=C6)C(=O)/C=C/C7=CC=C(C=C7)Br)C)C)(C)C	-	159.3 ± 13.6
	1b	CC1(C)C(CC[C@]2(C)[C@@]3([H])CC=C4[C@]5([H])CC(C)(C)CC[C@@](C(OC6=CC=C(C(/C=C/C7=CC=CO7)=O)C=C6)=O)5CC[C@](C)4[C@@](C)3CC[C@@]12[H])=O	-	3.2 ± 0.2
	1c	CC1(C)C(CC[C@]2(C)[C@@]3([H])CC=C4[C@]5([H])CC(C)(C)CC[C@@](C(OC6=CC=C(/C=C/C(C7=CC=CC=C7)=O)C=C6OC)=O)5CC[C@](C)4[C@@](C)3CC[C@@]12[H])=O	-	259.2 ± 14.1
	1e	CC1(C)C(CC[C@]2(C)[C@@]3([H])CC=C4[C@]5([H])CC(C)(C)CC[C@@](C(OC6=CC(/C=C/C(C7=CC=C(Cl)C=C7)=O)=CC=C6OC)=O)5CC[C@](C)4[C@@](C)3CC[C@@]12[H])=O	-	147.7 ± 4.9
	2a	O=C(OC1=CC=C(C=C1)C(/C=C/C2=CC=C(OC)C=C2)=O)[C@]34CCC(C)(C[C@]3(C5=CC[C@@]6([C@]7(CC(C(C=CC=C8)=C8N9)=C9C(C)([C@@]7(CC[C@]6([C@@]5(CC4)C)C)[H])C)C)[H])[H])C	-	52.7 ± 6.8
	2b	O=C(OC1=C(OC)C=C(C=C1)C(/C=C/C2=CC=C(OC)C=C2)=O)[C@]34CCC(C)(C[C@]3(C5=CC[C@@]6([C@]7(CC(C(C=CC=C8)=C8N9)=C9C(C)([C@@]7(CC[C@]6([C@@]5(CC4)C)C)[H])C)C)[H])[H])C	-	125.6 ± 10.7
	3b	CC1(C)C(C=C[C@]2(C)[C@@]3([H])CC=C4[C@]5([H])CC(C)(C)CC[C@@](C(OC6=CC=C(C(/C=C/C7=CC=C(OC)C=C7)=O)C=C6)=O)5CC[C@](C)4[C@@](C)3CC[C@@]12[H])=O	-	218.7 ± 1.5
	3c	CC1(C)C(C=C[C@]2(C)[C@@]3([H])CC=C4[C@]5([H])CC(C)(C)CC[C@@](C(OC6=CC=C(C(/C=C/C7=CC=CC=C7)=O)C=C6)=O)5CC[C@](C)4[C@@](C)3CC[C@@]12[H])=O	-	210.2 ± 1.7
	3d	CC1(C)C(C=C[C@]2(C)[C@@]3([H])CC=C4[C@]5([H])CC(C)(C)CC[C@@](C(OC6=CC=C(C(/C=C/C7=CC=CO7)=O)C=C6)=O)5CC[C@](C)4[C@@](C)3CC[C@@]12[H])=O	-	76.9 ± 4.7
	3e	CC1(C)C(C=C[C@]2(C)[C@@]3([H])CC=C4[C@]5([H])CC(C)(C)CC[C@@](C(OC6=CC=C(C(/C=C/C7=CC=CS7)=O)C=C6)=O)5CC[C@](C)4[C@@](C)3CC[C@@]12[H])=O	-	13.5 ± 1.5
	4a	O=C([C@]12CCC(C)(C)C[C@@]1([H])C3=CC[C@]4([H])[C@@]5(C)CC=CC(C)(C)[C@]5([H])CC[C@@]4(C)[C@]3(C)CC2)OC6=CC=C(C(/C=C/C7=CC=CC=C7Cl)=O)C=C6	-	30.8 ± 1.4
	4b	O=C([C@]12CCC(C)(C)C[C@@]1([H])C3=CC[C@]4([H])[C@@]5(C)CC=CC(C)(C)[C@]5([H])CC[C@@]4(C)[C@]3(C)CC2)OC6=CC=C(C(/C=C/C7=CC=C(OC)C=C7)=O)C=C6	-	98.9 ± 4.0
	4c	O=C([C@]12CCC(C)(C)C[C@@]1([H])C3=CC[C@]4([H])[C@@]5(C)CC=CC(C)(C)[C@]5([H])CC[C@@]4(C)[C@]3(C)CC2)OC6=CC=C(C(/C=C/C7=CC=CC=C7)=O)C=C6	-	31.8 ± 2.2
	4d	O=C([C@]12CCC(C)(C)C[C@@]1([H])C3=CC[C@]4([H])[C@@]5(C)CC=CC(C)(C)[C@]5([H])CC[C@@]4(C)[C@]3(C)CC2)OC6=CC=C(C(/C=C/C7=CC=CO7)=O)C=C6	-	30.5 ± 1.4
	4e	O=C([C@]12CCC(C)(C)C[C@@]1([H])C3=CC[C@]4([H])[C@@]5(C)CC=CC(C)(C)[C@]5([H])CC[C@@]4(C)[C@]3(C)CC2)OC6=CC=C(C(/C=C/C7=CC=CS7)=O)C=C6	-	14.2 ± 0.7
	4f	O=C([C@]12CCC(C)(C)C[C@@]1([H])C3=CC[C@]4([H])[C@@]5(C)CC=CC(C)(C)[C@]5([H])CC[C@@]4(C)[C@]3(C)CC2)OC6=CC=C(/C=C/C(C7=CC=CC=C7)=O)C(OC)=C6	-	163.8 ± 8.0
	5a	O=C([C@]12CCC(C[C@@]1([H])C3=CC[C@]4([H])[C@@]([C@@](CC[C@@]4(C)[C@]3(C)CC2)(C(C)=C)[H])(C)CCC#N)(C)C)OC5=CC=C(C(/C=C/C6=CC=C(C=C6)OC)=O)C=C5	-	48.0 ± 3.7
	5b	O=C([C@]12CCC(C[C@@]1([H])C3=CC[C@]4([H])[C@@]([C@@](CC[C@@]4(C)[C@]3(C)CC2)(C(C)=C)[H])(C)CCC#N)(C)C)OC5=CC=C(C(/C=C/C6=CC=CC=C6)=O)C=C5	-	20.4 ± 0.8
	5c	O=C([C@]12CCC(C[C@@]1([H])C3=CC[C@]4([H])[C@@]([C@@](CC[C@@]4(C)[C@]3(C)CC2)(C(C)=C)[H])(C)CCC#N)(C)C)OC5=CC=C(C(/C=C/C6=CC=CO6)=O)C=C5	-	4.1 ± 0.2
	5d	O=C([C@]12CCC(C[C@@]1([H])C3=CC[C@]4([H])[C@@]([C@@](CC[C@@]4(C)[C@]3(C)CC2)(C(C)=C)[H])(C)CCC#N)(C)C)OC5=CC=C(C(/C=C/C6=CC=CS6)=O)C=C5	-	11.5 ± 1.0
	5e	O=C(OC1=CC=C(C=C1)/C=C/C(C2=CC=CC=C2)=O)[C@]34CCC(C)(C[C@]3(C5=CC[C@@]6([C@@](CCC#N)([C@@]([H])(CC[C@]6([C@@]5(CC4)C)C)C(C)=C)C)[H])[H])C	-	33.9 ± 0.4
	6a	CC(C)(C[C@]1(C2C(C[C@@]3([C@@](CCC(OC4=CC=C(C(/C=C/C5=CC=C(OC)C=C5)=O)C=C4)=O)([C@@]([H])(CC[C@]3([C@@]2(CC6)C)C)C(C)=C)C)[H])=O)[H])CC[C@@]16C(OC)=O	-	10.8 ± 0.9
	6b	CC(C)(C[C@]1(C2C(C[C@@]3([C@@](CCC(OC4=CC=C(C(/C=C/C5=CC=CO5)=O)C=C4)=O)([C@@]([H])(CC[C@]3([C@@]2(CC6)C)C)C(C)=C)C)[H])=O)[H])CC[C@@]16C(OC)=O	-	8.1 ± 0.7
	6c	CC(C)(C[C@]1(C2C(C[C@@]3([C@@](CCC(OC4=CC=C(C(/C=C/C5=CC=CS5)=O)C=C4)=O)([C@@]([H])(CC[C@]3([C@@]2(CC6)C)C)C(C)=C)C)[H])=O)[H])CC[C@@]16C(OC)=O	-	15.5 ± 1.0
	Oleanolic acid (PC)		-	102.3 ± 2.4
	Acarbose (PC)		-	>300
Hu et al., 2012 [9]			IC_50_ (µM)	
Chalcone derivative/ Cleistocalyx operculatus	2',4'-dihydroxy-6'-methoxy-3',5'-dimethylchalcone	CC1=C(C(=C(C(=C1O)C(=O)/C=C/C2=CC=CC=C2)OC)C)O	43	NI
	acarbose		1.64	-

Inhibitory activities against α-amylase and α-glucosidase of chalcones

Chalcones With Basic Structural Framework

Many chalcone derivatives isolated from herbs have shown inhibitory activity on the enzymes α-amylase and α-glucosidase. Two chalcone derivatives isolated from *Dracaena cinnabari* include 2',4'-dihydroxychalcone (6), 4,4'-dihydroxy-2'-methoxychalcone (7) had α-glucosidase inhibitory activity with IC_50_ values of 389.6 and 270.2 μM, respectively. Chalcone 7 was stronger than chalcone 6, that revealed that the activity could be strenghened by the oxygenation pattern [5]. *Bauhinia pulla*, a plant of the Fabaceae mainly distributed in Thailand, contains anti-diabetic ingredients with chalcone framework, namely 3,2’,4’-trihydroxy-4-methoxychalcone, and 4-methyl ether isoliquiritigenin. 3,2’,4’-trihydroxy-4-methoxychalcone was 6-fold less α-glucosidase inhibitory activity than 4-methyl ether isoliquiritigenin, despite the former possesses more hydroxyl groups. IC_50_ values of these two compounds showed a weaker effect than standard acarbose (124.11 µg/mL) [6].

Among chalcones isolated from *Humulus lupulus*, 3′-prenylchalconaringenin and 3′-geranylchalconaringenin showed its dual inhibition against α-glucosidase and α-amylase. 3′-geranylchalconaringenin demonstrated an inhibitory effect on α-glucosidase 50-fold higher than acarbose (IC_50_ = 1.08 μM vs 51.30 μM, respectively) and also exhibited moderate inhibition against α-amylase (IC_50_ = 20.46 μM). Meanwhile, 3′-prenylchalconaringenin and chalconaringenin showed their effect 20-fold weaker than 3′- geranylchalconaringenin, however, its inhibition against α-glucosidase was still more effective than acarbose. Nevertheless, the inhibition of α-amylase of these three compounds was weaker than the positive control [7].

To discover antidiabetic natural ingredients on the α-glucosidase target, sixteen diarylheptanoidchalcones including katsumadainols A1−A16 (1-16), 13 similar known compounds (17-29) that originated from *Alpinia katsumadai* were isolated. Most compounds showed evidently inhibition against α-glucosidase as their IC_50_ values ranged from 2.9 and 29.5 µM, which were 6 to 59-fold stronger than acarbose (IC_50_ of 170.9 µM) [8]. 2',4'-dihydroxy-6'-methoxy-3',5'-dimethylchalcone (DMC), a compound isolated and purified from the dried flower buds of *Cleistocalyx operculatus* (Roxb.) Merr. et Perry (Myrtaceae), was studied *in vitro* and showed significant non-competitive inhibition (IC_50_ = 43 μM) of pancreatic α-amylase [9]. The synthesized chalcones also showed α-amylase and α-glucosidase inhibitory activity.

Chalcone 4 (butein) with C-2’-hydroxy, C-4’-hydroxy on the ring A and at C-3-hydroxy, C-4-hydroxy on the ring B gave the strongest efficiency against α-amylase. Most chalcones that had chloro-substitutent exhibited weak inhibition against α-amylase. The strongest inhibitor was chalcone 4 (butein) with IC_50_ value of 21 ± 2 μM followed by chalcone 21 (IC_50_ = 53 ± 1 μM) and chalcone 26 (IC_50_ = 87 ± 3 μM). Chalcone 4 (butein) was the most effective compound with an IC_50_ value 17-fold lower than that of acarbose with IC_50_ value of 357 ± 25 μM, which was the positive control [3].

In the series of synthetic chalcone derivatives of Cai et al. (2017) [10], compounds 1c, 1e, and 1f were affected by the number of MeO groups, thus, having weak α-glucosidase inhibitory activities compared to 1-deoxynojirimycin (IC_50_ of 21.3 ± 8.7µM). In contrast, the position of hydroxyl groups in the chalcone structure played a critical role in the inhibition of α-glucosidase. Ring A with 4-hydroxyl would have a stronger inhibitory effect than that with 3-hydroxyl group, proven by the IC_50_ value of compound 2c (13.4 ± 2.7 µM) compared to compound 2d (42.0 ± 6.0 µM). Nevertheless, a hydroxyl group added on C-3 position of the ring A of the compound 2b (IC_50_ = 35.2 ± 0.2 µM) enhanced the inhibition against α-glucosidase of compound 2f (IC_50_ =15.6 ± 2.8 µM). Besides, the chalcone derivative having the ring B with the 2’-hydroxyl group gave a stronger inhibitory effect than the 3’-hydroxyl group (compound 2b vs 2c) [10].

Thirteen synthesized chalcones (1-13) were primarily tested for their inhibition against α-amylase *in vitro*. All compounds were found to be effectively against α-amylase compared to the positive control (acarbose) with an IC_50_ value of 1.04 ± 0.3 µM. Their IC_50_ values ranged from 1.25 ± 1.05 to 2.40 ± 0.09 µM. The dominant compounds were 4 and 10. SMe and OMe-substituents were mainly responsible for the inhibitory effect of chalcones, as examined by the structure-activity relationship [11]. Xanthohumol showed its inhibition against α-glucosidase (IC_50_ = 8.8 µM) as a reversible and non-competitive inhibitor. Using caco-2 cell monolayers model, the compound prevented the separation of glucose from maltose in the apical side of these cells [12]. The presence of sulfonamide group on chalcone as compounds 2a-2h led to a moderate inhibition against α-glucosidase in comparison with acarbose (IC_50_ = 0.95 ± 0.28 μM). The inhibitory effect decreased when the size of substituents increased on para-position on the ring B. The compound 2a was the strongest inhibitor with IC_50_ of 4.2 ± 0.21 μM, then followed by compound 2b (IC_50_ = 5.4 ± 0.10 μM), compound 2c (IC_50_ = 6.1 ± 0.56 μM), and compound 2d (IC_50_ = 8.1 ± 0.61 μM) [11]. Compounds 2a-2h were evaluated for their inhibition against α-amylase. Generally, sulfonamide group would diminish the inhibitory activities of compounds [13]. Nine synthesized chalcones were experimented regarding their inhibition against α-glucosidase in a study. Their resulting IC_50_ values indicated their significant efficiency. However, among those compounds, only chalcone 34 (2'-hydroxychalcone) (IC_50 _= 15.0 µM) and chalcone 35 (4-amino-4'-methoxychalcone) (IC_50_ = 20.2 µM) demonstrated their promising anti-diabetic effect compared to positive control deoxynojirimycin (IC_50_ = 3.49 µM) [14].

Among synthesized aminochalcones and sulfonamide chalcones in Seo et al. (2005), only chalcone 15, 16 and sulfonamide chalcones (17-20) exhibited their remarkable inhibition against α-glucosidase. Particularly, the inhibitory effect of sulfonamide chalcone 20 was 150-fold stronger than acarbose (IC_50_ = 60.8 μM). For the inhibition of α-amylase, only sulfonamide chalcone (17-20) showed strong inhibition [15]. The compounds 2, 6, 13, 17, 18, 19, 21, and 22 showed their potential inhibition against α-glucosidase in comparison with the positive control deoxynojirimycin. Among these, compound 2 (IC_50_ = 15 ± 0.14) and compound 21 (IC_50 _= 20 ± 1.42 µM) were the most potential candidates [16].

Lin et al. (2020) synthesized five chalcones. Amongst these, only chalcone 1 (2'-bromoethoxychalcone) and 2 (2'-bromopropoxychalcone) actively inhibited against α-glucosidase at 10 mmol/L. Chalcone 3 (2'-bromo butoxychalcone) only showed its effect merely at the higher concentration of 20 mmol/L [17]. Five synthesized derivatives of oleanolic acid chalcone were evaluated for their inhibitory ability against α-glucosidase. Among these, compound 2b (IC_50 _= 47.5 μM) exhibited the strongest inhibition against α-glucosidase with 8-fold more effectiveness than acarbose. Thus, the development of α-glucosidase inhibitors that had a scaffold of oleanolic acid-chalcone could bring many innovative approaches for T2D patients [18].

Most synthesized oleanolic acid derivatives chalcones reported by Tang et al. (2014) exhibited moderate inhibitory activity against α-glucosidase [19]. The para-position of the secondary amine (compound 3c) and the presence of electron donating groups (compound 3e) were suggested to affect the inhibitory ability. Compound 3c was a strong inhibitor with an IC_50_ value of 1.04 ± 0.19 μM. This proved that chalcone sulfonamide had a large potential as α-glucosidase inhibitor in the treatment of diabetes [20]. Chalcones 5’-prenylated (1) and 5’-geranylated chalcone (14) were both a bit more effective than acarbose. It should be noted that 3’5’-diprenylated and digeranylated chalcones had a remarkeable inhibitory effect. Of which, the digeranylated chalcone (16) was the best inhibitor against α-glucosidase (IC_50_ = 0.9 μM) [21].

Among the synthesized chalcone triazoles in Chinthala et al. (2015), compounds 4m, 4p, and 4s were promising candidates to inhibit α-glucosidase [22]. Testing the inhibition against α-amylase of compound 3 was conducted as described in the test kit, acarbose (IC_50_ = 1.03 ± 0.05 μM) was used as a positive control. The location of fluo-groups on the ring B of the chalcone scaffold resulted in different action manners of 5-styryl-2-aminochalcones (3a-h). The presence of 50-styryl in 20-amino-3-fluorochalcone scaffold markedly decreased the effect of the styryl-chalcone compound (3a) with IC_50 _of 10.7 ± 0.21 μM. On the other hand, 20-amino-50-styryl-4-fluorochalcone isomer (3e) showed significant inhibition against α-amylase with the IC_50 _value of 1.6 ± 0.52 μM. Compounds 20-amino-50-(4-fluorostyryl)-3-fluorochalcone (3b; IC_50_ = 15.6 ± 0.60 µM) and 20-amino-50-(4-fluorostyryl)-4-fluorochalcone isomer (3f; IC_50_ = 9.5 ± 0.41 µM) also shared this tendency. Besides, 20-amino-50-(4-chlorostyryl)-3-fluorochalcone (3c) improved the inhibition against the enzyme with IC_50_ = 2.5 µM. Its isomer, 20-amino-50-(4-chlorostyryl)-4-fluorochalcone (3g), also shared this pattern with IC_50_ = 1.7 ± 0.25 µM, respectively. 20-amino-50-(4-methoxystyryl)-3-fluorochalcone (3d) (IC_50_ = 7.0 ± 0.45 µM) and also its isomer (3h) (IC_50_ = 7.6 ± 0.20 µM) had been proved as moderate inhibitors against α-amylase [23]. The combination of 50-styryl and 20-amino-3-fluorochalcone led to a significant decrease in the inhibition of compound 3a (IC_50_ = 17.8 ± 0.32 µM). In addition, the compound 3b (having 50-(4-fluorostyryl) group and inhibition 20-amino-3-fluorochalcone scaffold) had its inhibition against α-glucosidase improved, as its IC_50_ values of 6.1 ± 0.24 µM. A derivative of compound 3c with a substituent of 50-(4-chlorostyryl) revealed a slightly improved effect (IC_50_ = 12.6 ± 0.31 µM). A combination of the hydrophobic 50-(4-methoxystyryl) group and the 20-amino-3-fluorochalcone group in the scaffold of compound 3 likely improved the inhibition against α-glucosidase (IC_50_ = 9.4 ± 0.50 µM). The compound 3e, with its structure having the 50-styryl group and the 20-amino-4-fluorochalcone group showed the highest inhibition among derivatives of 5-styryl-2-aminochalcone scaffold with IC_50_ of 5.1 ± 0.61 µM. The presence of 50-(4-fluorostyryl) and 20-amino-4-fluorochalcone groups increased the inhibitory effect of the compound 3f (IC_50_ = 6.9 ± 0.37 µM). Moreover, the 20-amino-4-fluorochalcone scaffold with an electron-withdrawing group 50-(4-chlorostyryl) significantly decreased the effect of the compound 3g (IC_50_ = 19.2 ± 0.47 µM). The stronger inhibition against α-glucosidase of compound 3h, which had a strong electron-donating group 4-methoxystyryl, was also observed with IC_50 _value of 10.5 ± 0.18 µM. Nevertheless, the isomer of the compound 3d was slightly more active [23]. Sixteen synthesized chalcones were examined *in vitro* in terms of their inhibition against α-amylase from the porcine pancreas. All chalcones showed better inhibition than standard control acarbose (IC_50_ values ranging from 1.25 ± 1.05 to 2.40 ± 0.09 μM vs IC_50_ = 1.34 ± 0.3 μM). Among these, compounds 2-4, 6, 9, and 12-15 revealed modest to good inhibitory effect as their IC_50 _values ranged from 1.47 ± 0.481 to 1.89 ± 0.126 μM [24].

Azachalcones

Of the twenty-seven synthesized azachalcone derivatives (3-29), compound 3, compound 5, compound 6, compound 16, and compound 28 were strong α-amylase and α-glucosidase inhibitor. Their IC_50 _are presented in Table 2. Among these, compound 3, which had the 3-acetyl pyridine group and 2-methoxy-aryl group in its structure, was the most potent inhibitor against these enzymes [25].

Bis-chalcones

Most bis-chalcones showed stronger inhibition against α-glucosidase than standard compound 1-deoxynojirimycin (IC_50_ = 21.3 ± 8.7 µM). Bis-chalcone 2k was the strongest inhibitor, as its IC_50_ value was 1.0 ± 0.1 µM. Compounds 2g, 2j, and 2l were the strongest non-competitive inhibitors, as shown by the kinetic analysis [7]. All five bis-chalcones synthesized by Bale et al. (2018)(14-18) were significant α-amylase inhibitor *in vitro*, as its effect (IC_50_ values ranging from 1.63 ± 0.18 to 2.4 ± 0.09 µM) was comparable to standard control acarbose (IC_50_ = 1.04 ± 0.3 µM) [10].

Chalcone Analogs

Cinnamylidene acetophenone (41) (an analog of chalcone) with two double bones that linking ring A and ring B showed α-glucosidase inhibitory activity with IC_50_ of 41 ± 1 µM. The efficiency was 9-fold better than positive control acarbose (IC_50_ = 357 ± 25 µM) [3].

Chalcone Oximes

Fandaklı et al. (2018) reported the α-glucosidase inhibitory activity of synthesized chalcone oximes (2a-2k). Amongst tested compounds, compounds 2a and 2b showed more effective activity against α-glucosidase with IC_50_ values of 1.61 ± 0.16 µM and 3.36 ± 0.58 µM [26].

Coumarin-Chalcone Derivatives

Of the 22 coumarin-chalcone derivatives (3a-3v) synthesized by Hu et al. (2022), compounds 3j, 3q, and 3t were significant α-glucosidase inhibitors, as their IC_50 _values were 30.30 ± 2.53, 29.74 ± 2.68, 24.09 ± 2.36 μM, respectively, which were all stronger than acarbose [27].

Cyclohexane Chalcones

Three cyclohexenyl chalcones from *Boesenbergia rotunda* included 3 (panduratin A), 4 (isopanduratin A) and 7 (hydroxypanduratin A) showed α-glucosidase inhibition. Their IC_50 _value was 12.7 ± 1.3, 7.5 ± 0.6, 4.6 ± 0.4 µM, respectively. The inhibitory activity of these compounds was many-fold stronger than acarbose (1155.5 ± 23.0) [28].

Dihydrochalcones

4,4'-dihydroxy-2-methoxydihydrochalcone (5), a dihydrochalcone from *Dracaena cinnabari, *has a weaker α-glucosidase inhibitory activity than chalcones with basic structural framework as 2',4'-dihydroxychalcone (6) and 4,4'-dihydroxy-2'-methoxychalcone (7) [5]. Dracidione (IC_50_ = 40.2 µg/ml) is a new chalcone-dihydrochalcone dimer from *Dracaena cinnabari* with C-linked that significantly inhibited α-glucosidase. Its effect was only a bit weaker than acarbose (IC_50_ = 30.5µg/ml) [29]. Meanwhile, dihydrochalcone glucosides showed stronger α-glucosidase inhibitory activity. Six dihydrochalcone glucosides (5-9) from *Balanophora tobiracola* had inhibitory activity against α-glucosidase at a lower concentration (IC_50_ values ranged from 0.4 to 1.8 μg/ml) [30]. A compound extracted from *Boesenbergia rotunda* named 2',4',6'-trihydroxydihydrochalcone had the inhibitory activity against α-glucosidase with IC_50_ value of 32.0 ± 2.2 µM many-fold lower than acarbose (IC_50_ = 1155.5 ± 23.0 µM) [28]. For α-amylase inhibitory activity, among six synthesized dihydrochalcones, compound 3e (IC_50_ = 23.17 μg/ml) showed the strongest activity against α-amylase, followed by compounds 3a (IC_50_ = 25.70 μg/ml) and compound 3f (IC_50_ = 48.17 μg/ml). In comparison with acarbose, which had IC_50_ value of 891.25 μg/ml, all these compounds were more effective [31].

Flavanone-Coupled Chalcones

Biflavones (9,12-14) from *Boesenbergia rotunda* showed marked inhibition against α-glucosidase. Among those, flavone-coupled chalcones 9, 12, 13 (IC_50_ ranged from 1.3 to 3.4 μM) were found to be 3-10 folds more active than normal chalcone and even more effective than acarbose (IC_50_ = 1155.5 ± 23.0 μM) [28]. 

Potential Toxicity of Chalcone Derivatives in Humans

Natural chalcones were considered relatively non-toxic compounds [32]. The prenylated chalcone from *Humulus lupulus* was known as xanthohumol to have anti-inflammatory effects in healthy humans if used in low doses achievable through the diet, it was safe and well tolerated by healthy adults at doses of 24 mg per day [33,34]. Licochalcone A was an ingredient in a moisturizer that has been shown to be effective and safe for people with mild to moderate acne [35]. Hydroxysafflor Yellow A for injection was safe and well-tolerated at all doses for treating acute ischemic stroke patients with blood stasis syndrome [36].

However, the toxicity of new synthetic chalcones were not well known. In addition, antidiabetic drugs that inhibit α-amylase and α-glucosidase enzymes known as acarbose have been reported to have side effects such as stomach discomfort, gas, bloating and diarrhea [37]. Some chalcone derivatives have been shown to be more active in vitro than acarbose. Therefore, concern about the toxicity and safety of these compounds was necessary.

Several studies on chalcones toxicity have been reported. 3-(4,5-dimethylthiazol-2-yl)-2,5-diphenyltetrazolium bromide (MTT) assay on normal monkey kidney cells (Vero cells) and adenocarcinomic human epithelial cells (A549 cells) showed that 5-styryl-2-sulfonamidochalcones (2f and 2h) had no cytotoxic effect on the Vero cells and narrowed the toxicity on A549 cells [13]. Predictive toxicity studies have shown that chalcone triazoles are not mutagenic and are not irritating to the skin and eyes. In addition, their toxicity dose range is also predicted in silico with rat oral LD50 from 0.64 g/kg to 7.94 g/kg [22]. Meanwhile, chalcones and chalcone analogues in the study by Lee et al. (2014) showed embryotoxicity of zebrafish resulting in muscle defects [38].

Computational methods for chemical toxicity prediction can also be used to select potential candidates for further study or to design a series of new structures with reduced toxicity.

## Conclusions

This narrative review presented chalcones and their derivatives as potential scaffolds with chemical characteristics that strongly affect α-amylase and α-glucosidase. Some compounds showed better efficacy in vitro than standard controls, suggesting approaches for developing new drugs to manage diabetes. Although the effects of chalcone derivatives were mentioned in several studies, the toxicity of new synthetic chalcones are not well known. Considerably more work will need to be done to determine the potential effects of Chalcones derivatives in humans. The results demonstrated positive findings that support the potential use of these candidates for discovering new anti-diabetic remedies in the years to come.
